# The PRAGMATIC pathway ‐ PRostate cancer diAGnosis and MAnagement Triage In Clinical care

**DOI:** 10.1111/bju.70191

**Published:** 2026-02-26

**Authors:** Abhishek Sharma, Teresa Campbell, Sagar Kanabar, Hannah Soanes, Ganesh Sathanapally, Anthony Bates, Rincy John, Charlotte Adams, Aisling Brassill, Bryony Lennon, Sanjay Sinha, Louise Flaxman, Vanessa von Hasseln, Philip Camilleri, Ami Sabharwal, Philip Charlton, Gerard Andrade, Mark Tuthill, Andrew Protheroe, Alastair D. Lamb, Tom Leslie, Aaron Leiblich, Francisco Lopez, Clare Verrill, Fergus Gleeson, Ruth MacPherson, Freddie C. Hamdy, Richard Bell, Richard J. Bryant

**Affiliations:** ^1^ Department of Urology Oxford University Hospitals NHS Foundation Trust, Churchill Hospital Oxford UK; ^2^ Oxford Transplant Centre Oxford University Hospitals NHS Foundation Trust, Churchill Hospital Oxford UK; ^3^ Department of Oncology Oxford University Hospitals NHS Foundation Trust, Churchill Hospital Oxford UK; ^4^ Department of Radiology Oxford University Hospitals NHS Foundation Trust, Churchill Hospital Oxford UK; ^5^ Department of Urology NHS Lothian, Western General Hospital Edinburgh UK; ^6^ Quality Improvement Team Oxford University Hospitals NHS Foundation Trust, John Radcliffe Hospital Oxford UK; ^7^ Department of Cellular Pathology Oxford University Hospitals NHS Foundation Trust, John Radcliffe Hospital Oxford UK; ^8^ Nuffield Department of Surgical Sciences University of Oxford Oxford UK

**Keywords:** prostate cancer, investigation, faster diagnosis, risk‐stratification, triage, quality improvement

## Abstract

**Objectives:**

To investigate whether nurse navigator‐led triaging of high‐risk patients may reduce prostate cancer (PCa) diagnosis and treatment times using an in‐house bespoke PRostate cancer diAGnosis and MAnagement Triage In the Clinial care pathway (PRAGMATIC) triaging system, as locally advanced/metastatic disease should be diagnosed and treated rapidly, and UK targets allow 28 days for diagnosis, and 62 days to commence treatment.

**Patients and Methods:**

We reviewed diagnosis and treatment timelines for patients undergoing 28/62‐day investigation for suspected PCa at a tertiary unit in a 3‐month period (2022). We then introduced nurse navigator‐led triaging of urgent referrals and evaluated a subsequent 3‐month period (2024), with streamlining for rapid investigation and treatment based on prostate‐specific antigen, magnetic resonance imaging (MRI) staging, and histology. We hypothesised nurse navigator‐led triaging would improve investigation and treatment times for high‐risk localised, or locally advanced, or metastatic PCa.

**Results:**

A total of 165 and 327 consecutive patients were on the 28/62‐day PCa pathway in the pre‐ (2022) and post‐nurse navigator‐led (2024) triaging periods, respectively. The median time from referral to first appointment (8 vs 4 days), MRI (12 vs 6 days), MRI result (26 vs 17 days), biopsy decision (25 vs 16 days), biopsy procedure (48 vs 22 days), biopsy result communication (64 vs 44 fays), and prostate‐specific membrane antigen positron emission tomography computed tomography staging scan (87 vs 56 days) was reduced following nurse navigator triaging of high‐risk cases (all *P* < 0.001). The median time from referral to treatment for Gleason Grade Group ≥3, or T3, or ≥N1, or ≥M1 disease (104 vs 70 days; 49/75 [65.3%] vs 72/128 [56.3%] patients), and for M1b disease (47 vs 27 days; 15/75 [20%] vs 32/128 [25%] patients), was reduced (*P* < 0.05).

**Conclusions:**

Nurse navigator‐led triaging and stratification of the most clinically urgent suspected PCa cases was associated with improved imaging, biopsy diagnosis, and treatment times for the highest‐risk patients.

**Abbreviations**
ADT: androgen deprivation therapy
AI: artificial intelligence
ANP: advanced nurse practitioner
AS: active surveillance
COVID‐19: coronavirus disease 2019; 28/62‐day, 28 days for diagnosis and 62 days to commence treatment
EAU: European Association of Urology
FDS: Faster Diagnosis Standard
LATP: local anaesthetic transperineal
MDT: multidisciplinary team
mpMRI: multiparametric MRI
PCa: prostate cancer
PET: positron emission tomography
PRAGMATIC: PRostate cancer diAGnosis and MAnagement – Triage In the Clinical care
PSMA: prostate‐specific membrane antigen
QIP: Quality Improvement Project

## Introduction

Prostate cancer (PCa) is the commonest malignancy diagnosed in men in the UK, with 52 300 new cases, and 12 200 disease‐specific deaths, annually [1]. In all, 12% of patients in England presented with metastatic disease in 2022 [2]. Guidelines for urgent referral for investigation of suspected PCa, and diagnosis and treatment targets, were introduced in England in 2000 [3] to improve clinical outcomes. Faster Diagnosis Standard (FDS) targets have been introduced aiming to diagnose, or exclude, PCa within 28 days, and to commence treatment within 31 days of diagnosis [4, 5]. Whilst these arbitrary targets may lead to reduced time‐to‐diagnosis and time‐to‐treatment, there is no evidence of improved PCa‐specific survival from meeting these targets [6]. The PCa investigation pathway is complex, with pre‐biopsy multiparametric MRI (mpMRI) [7, 8], avoidance of biopsy if negative mpMRI [7, 8, 9], targeted biopsy [10] via TRUS or local anaesthetic transperineal (LATP) [11], and prostate‐specific membrane antigen positron emission tomography computed tomography (PSMA‐PET‐CT) staging [12]. Management options have for localised disease include active surveillance (AS), radical surgery, radical radiotherapy (external beam or stereotactic ablative) with/without neoadjuvant androgen deprivation therapy (ADT), brachytherapy, and focal therapy [8]. Increased pathway complexity, absence of risk‐stratification within urgent referral criteria, and the need for patients to be informed of options at each step, risks patients with the most clinically significant disease (disease that needs commencement of urgent treatment, such as *de novo* locally advanced/metastatic cases) having rapidity of diagnosis and treatment compromised by resource use investigating less significant cases.

Arbitrary FDS 28/62‐day targets (UK targets allow 28 days for diagnosis, and 62 days to commence treatment, from initial referral) for localised PCa may not improve clinical outcomes. The Prostate testing for cancer and Treatment (ProtecT) trial in the UK demonstrated low clinical progression and mortality rates for clinically localised PCa at a median of 15 years follow‐up, regardless of whether patients were randomised to receive active monitoring, radical surgery or radical radiotherapy [13]. During the coronavirus disease 2019 (COVID‐19) pandemic, clinical priority for treating urological conditions was diverted away from PCa, instead focussing on bladder and renal cancer, and time‐critical benign conditions such as ureteric stone disease [14]. COVID‐19 required urologists, and policymakers such as BAUS, to prioritise conditions in need of rapid diagnosis and treatment. Focus has subsequently moved back towards the FDS pathway, such as the recent Rapid Assessment for Prostate Imaging and Diagnosis (RAPID) PCa diagnosis pathway [15]. However, whilst the aim of rapidly diagnosing all PCa cases is laudable, this may not accelerate delivery of care to those most in need and may divert resources away from other time‐critical urological conditions [16].

Clinicians recognise that high‐risk localised (such as those defined by the European Association of Urology [EAU] [8]), locally advanced, and metastatic PCa requires expedient investigation and treatment. However, it is pragmatic that low‐risk localised cases do not require rapid diagnosis or treatment. Very low‐risk PCa may not even need diagnosis, with AS being recommended, which may be lifelong. The FDS pathway referral criteria, and targets for diagnosis and treatment, are a ‘one size fits all’ approach, which do not facilitate prioritisation of potential high‐risk PCa cases at referral (such as those at highest risk of locally advanced or metastatic disease) nor during the clinical pathway of investigation and treatment. There is an unmet clinical need to deliver a more effective PCa diagnosis and management pathway, meeting urgent requirements for high‐risk cases, without detriment to low‐risk cases. We tested the hypothesis that introduction of protocolised nurse navigator‐led triaging would be associated with shorter times to diagnosis and treatment of high‐risk PCa cases at our institution.

## Patients and Methods

### Registration of Quality Improvement Programme

We registered an actionable review of the urgent PCa diagnosis and treatment pathway at Oxford University Hospitals NHS Foundation Trust, (OUHNHSFT), and undertook an internal Quality Improvement Project (QIP, reference 8381) to improve practice in this pathway for patients with the highest need for rapid diagnosis and treatment referred to our Institution. This process built on work in our Urology Department to improve implementation of the FDS pathway, such as evaluation of the pathway by advanced nurse practitioners (ANPs) and clinical and non‐clinical managers, recruitment of a nurse navigator and non‐clinical navigator to track and prioritise patients on the pathway based on need according to in‐house criteria, and focussed help from Cancer Improvement Team members and other stakeholders in the clinical pathway (including Radiology, Histopathology, and Clinical and Medical Oncology). This QIP aimed to allow the nurse navigator, alongside all engaged in the various steps of the urgent pathway, to triage the high‐risk cases for most rapid diagnosis and treatment, whilst simultaneously safeguarding against undue delay for low‐risk cases as an unintended consequence. The QIP was reviewed by all stakeholders in the delivery of the clinical pathway at our Institution, which was important as some specialties may need to slightly change practice to enable the proposed triaging protocol to be delivered effectively.

### Retrospective Review of Pre‐Nurse Navigator‐Led Pathway Timings

A retrospective review was conducted of all PCa‐naïve urgent patients on the 28/62‐day FDS pathway for suspected PCa in a historical 3‐month period (July, September, December 2022), this being prior to the implementation of nurse navigator‐led triaging of high‐risk patient referrals. These 3 months of 2022 were chosen to try and avoid seasonal variation in referral numbers, and to try an avoid the practical impact of supply difficulty for F18‐1007 PSMA tracer, which was an intermittent issue at the time. This retrospective analysis allowed us to understand timelines and bottlenecks in the pathway from referral to treatment, including for the high‐risk patients undergoing PSMA‐PET‐CT molecular imaging for staging, which may add delay to the pathway.

### Review of Post‐Nurse Navigator‐Led Pathway Timings

**Fig. 1:**
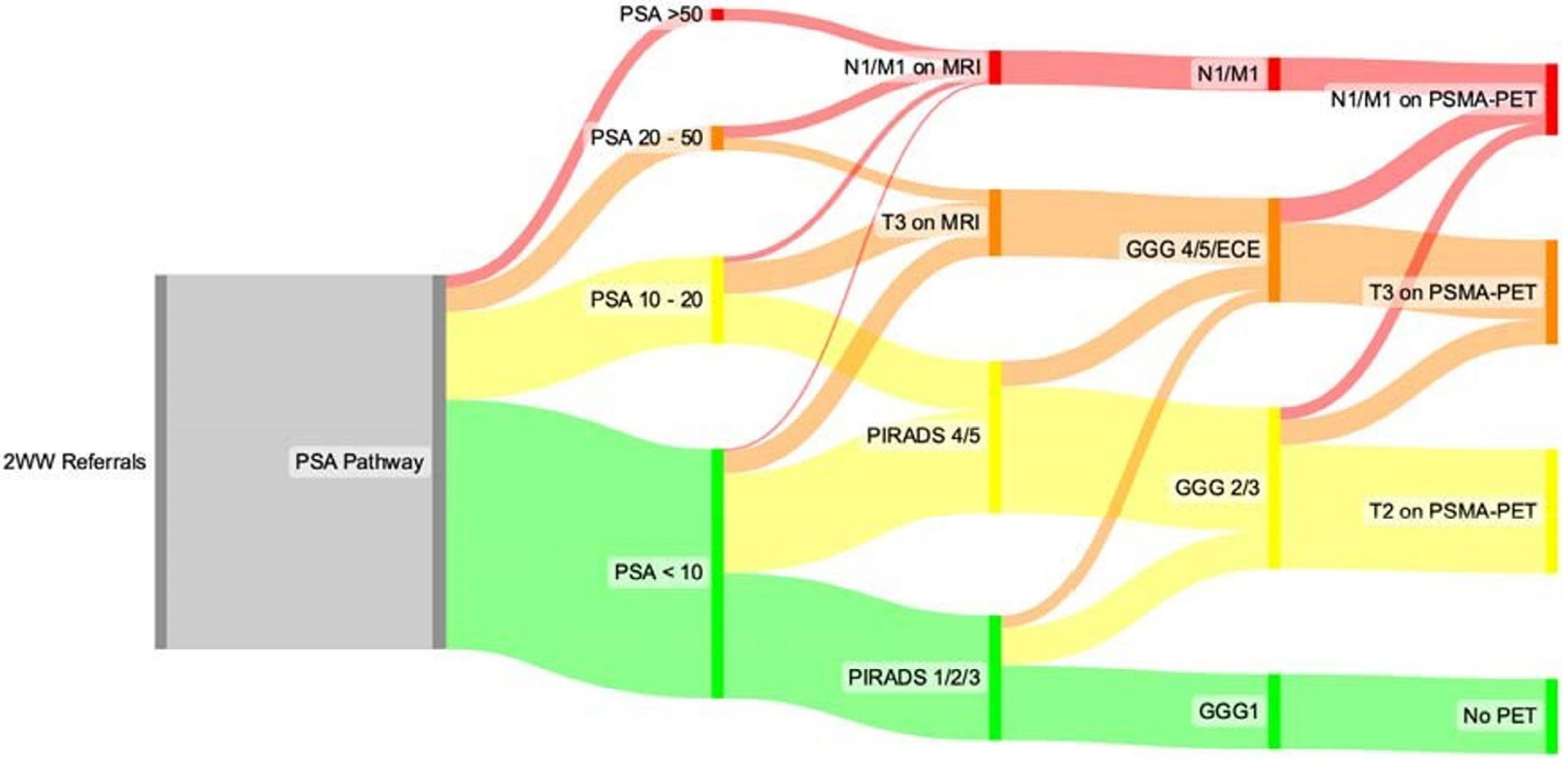
The PRAGMATIC streaming principles used in the 2‐week wait (2WW) suspected PCa pathway used following appointment of the nurse navigator. GGG, Gleason Grade Group; PIRADS, Prostate Imaging Reporting and Data System.

Following this retrospective evaluation of practice, we prospectively audited a 3‐month period (June, July, August 2024) following nurse navigator‐led implementation of internal PRostate cancer diAGnosis and MAnagement Triage In the Clinial care pathway (PRAGMATIC) triaging, this being based on risk‐stratification criteria such as the EAU risk groups for biochemical recurrence of localised and locally advanced PCa [7] (Table 1). The stratification process was dynamic at multiple steps in the pathway, including at the multidisciplinary team (MDT) review. Patients could be moved upwards or downwards into a different stream, dependent upon investigations at each node in the pathway. For example, an individual with a PSA level ≥50 ng/mL potentially has locally advanced or metastatic PCa and could be placed in the highest‐risk category. The PRAGMATIC stratification streaming criteria are shown in Fig. 1, and a visual detail of the nurse navigator‐led triaging of the clinical pathway for patients urgently referred with suspected PCa is shown in Fig. S1. The aim of this approach was to allow efficient triage of pre‐biopsy mpMRI, prostate biopsy appointments, PSMA‐PET‐CT and other staging investigations. Nurse‐led triaging of the pathway allowed assignment of patients to either ANP‐ or consultant‐led results clinics (Fig. S1), and to consultant‐led treatment discussion clinics, to prioritise a more rapid pathway for the highest‐risk patients, whilst simultaneously ensuring no patients are disadvantaged by the internal triaging process.

**Table 1:** Baseline demographics of all diagnosis‐naïve patients referred on the urgent suspected PCa pathway in the pre‐ and post‐nurse ‘navigator’ periods of analysis.

Variable	Pre‐navigator, *N* = 165	Post‐navigator, *N* = 327	*P*
Age, years, mean (median, range)	68.5 (69, 38–92)	69.3 (69, 40–95)	0.286*
PSA level, ng/mL, mean (median, range)	19.8 (7.4, 0.37–735)	28.5 (7.92, 0.55–1628)	0.224*
PSA level 0 to <20 ng/mL, *n/N* (%)	134/164 (81.7	272/321 (84.8)	
PSA level 20 to <100 ng/mL, *n/N* (%)	26/164 (15.9)	38/321 (11.8)	
PSA level ≥100 ng/mL, *n/N* (%)	4/164 (2.4)	11/321 (3.4)	
Lost to follow‐up/non‐compliant, *n*	1	6	

Patients were excluded from subsequent analyses if they did not comply with the pathway or if they were lost to follow‐up.

* Independent‐Samples Mann–Whitney *U* test.

### Statistical Analysis

All statistical analysis was undertaken using Statistical Package for the Social Sciences (SPSS) software (IBM Corp., Armonk, NY, USA), using the independent‐samples Mann–Whitney *U* Test for non‐normally distributed continuous data (e.g., patient age, PSA level, and time in days to each investigation or clinical appointment or commencement of treatment) or the Pearson chi‐square test for statistical analysis of categorical data (e.g., the proportions of patients receiving an investigation or appointment), comparing the pre‐ and post‐navigator groups on the clinical pathway, as indicated in the results tables. Data for the very few patients lost to follow‐up, or non‐compliant with investigation, was not included in the statistical analysis. Statistical significance is reported at the 5% significance level.

## Results

### Baseline Demographics and Referral Numbers

Baseline demographics of all diagnosis‐naïve patients listed on the FDS urgent suspected PCa pathway in the pre‐ and post‐nurse navigator 3‐month periods of analysis in 2022 and 2024 respectively are provided in Table 1. A small number of patients (one in the pre‐navigator period, and six in the post‐navigator period) were excluded from subsequent analyses if they did not comply with the FDS pathway or if they were lost to follow‐up. There was no significant difference in the median age at referral (69 years for each cohort, *P* = 0.286), or in the median PSA level (7.4 vs 7.92 ng/mL, *P* = 0.224), in the pre‐ and post‐nurse navigator cohorts. The PSA threshold distributions (PSA level <20, 20–<100, and ≥100 ng/mL) were similar between the two cohorts. A more patients were on the 28/62‐day suspected PCa pathway in the 3‐month post‐navigator period in 2024 (327) compared with the 3‐month pre‐navigator period in 2022 (165). This was consistent with there being a year‐on‐year rise in referral numbers, and with a higher number of patients being on the 28/62‐day suspected PCa pathway in the post‐navigator analysis year of 2024 (1610) compared with either 2023 (1319) or the pre‐navigator analysis year of 2022 (1128).

### Analysis of Times from Referral to each Specific Investigation on the Suspected PCa Pathway

Analysis of times to investigation, and features of these investigations, for patients referred on the urgent suspected PCa pathway in the pre‐ and post‐nurse navigator time periods is shown in Table 2. The median time to first clinic appointment upon receipt of the referral was significantly reduced from 8 to 4 days through use of the nurse navigator (*P* < 0.001), despite the number of referrals onto the pathway increasing from 165 to 327. Similar proportions of patients proceeded to MRI (139/164 [84.8%] pre‐ vs 280/321 [87.2%] post‐navigator, *P* = 0.638), were discharged for ‘PSA observation’ or ‘watchful waiting’ without biopsy (93/164 [56.7%] pre‐ vs 156/321 [48.6%] post‐navigator, *P* = 0.091), proceeded to prostate biopsy (84/164 [51.2%] pre‐ vs 162/321 [50.5%] post‐navigator, *P* = 0.875), were found to have biopsy‐confirmed PCa (68/84 [81%] pre‐ vs 118/162 [72.8%] post‐navigator, *P* = 0.16), and proceeded to PSMA‐PET‐CT for staging (31/68 [45.6%] pre‐ vs 55/118 [46.6%] post‐navigator, *P* = 0.852). The median time from referral to first appointment (8 vs 4 days), MRI (12 vs 6 days), MRI result (26 vs 17 days, where MRI performed), biopsy decision (25 vs 16 days, with these data including those patients in whom an MRI was contra‐indicated), biopsy procedure (48 vs 22 days), biopsy result to patient (64 vs 44 days), and PSMA‐PET‐CT staging scan (87 vs 56 days) was significantly reduced during the period of nurse navigator‐led triaging of highest‐risk cases, compared to the pre‐navigator period (*P* < 0.001 for each).

**Table 2:** Analysis of times to investigation, and features of these investigations, for patients referred on the urgent suspected PCa pathway in the pre‐ and post‐nurse ‘navigator’ time periods.

Variable	Pre‐navigator, *N* = 165	Post‐navigator, *N* = 327	*P*
Time to first appointment, days, mean (median, range)	9 (8, 2–24)	5 (4, 0–24)	**<0.001** *
Discharged for ‘PSA observation’, or ‘watchful waiting’ without biopsy, *n/N* (%)	93/164 (56.7)	156/321 (48.6)	0.091^†^
Proceeded to MRI, *n/N* (%)	139/164 (84.8)	280/321 (87.2)	0.638^†^
Time to MRI, days, mean (median, range)	14 (12, 2–49)	8 (6, 1–58)	**<0.001** *
Time to MRI result, days, mean (median, range)	29 (26, 6–95)	19 (17, 3–74)	**<0.001** *
Proceeded to biopsy, *n/N* (%)	84/164 (51.2)	162/321 (50.5)	0.875^†^
Time to decision regarding biopsy, days, mean (median, range)	28 (25, 2–95)	20 (16, 1–82)	**<0.001** *
Time to biopsy, days, mean (median, range)	50 (48, 13–189)	27 (22, 8–121)	**<0.001** *
TRUS biopsy, *n/N* (%)	56/84 (66.7)	132/162 (81.5)	
LATP biopsy, *n/N* (%)	28/84 (33.3)	24/162 (14.8)	
GATRUS biopsy, *n/N* (%)	0	1/162 (0.6)	
GATP biopsy, *n/N* (%)	0	5/162 (3.1)	
Benign/ASAP/PIN on biopsy, *n/N* (%)	16/84 (19)	44/162 (27.2)	0.16^†^
Biopsy detected prostate cancer, *n/N* (%)	68/84 (81)	118/162 (72.8)	0.16^†^
GGG1, *n/N* (%)	4/68 (5.9)	11/118 (9.3)	
GGG2, *n/N* (%)	27/68 (39.7)	49/118 (41.5)	
GGG3, *n/N* (%)	15/68 (22.1)	31/118 (26.3)	
GGG4, *n/N* (%)	9/68 (13.2)	10/118 (8.5)	
GGG5, *n/N* (%)	13/68 (19.1)	16/118 (13.6)	
Ungradable/ADT, *n/N* (%)	0	1/118 (0.8)	
Time to biopsy result, days, mean (median, range)	68 (64, 31–190)	48 (44, 20–145)	**<0.001** *
PSMA‐PET‐CT performed for staging, *n/N* (%)	31/68 (45.6)	55/118 (46.6)	0.852^†^
Time to PSMA‐PET‐CT, days, mean (median, range)	89 (87, 46–280)	68 (56, 38–316)	**<0.001** *
Lost to follow‐up/non‐compliant, *n*	1	6	

Bold values statistically significant at *P* < 0.001. ASAP, atypical small acinar proliferation; GA, general anaesthesia; GGG, Gleason Grade Group; PIN, prostatic intraepithelial neoplasia.

* Independent‐samples Mann–Whitney *U* test.

^†^ Pearson chi‐square.

### Analysis of Times from Referral to Commencement of Treatment on the Suspected PCa Pathway

**Fig. 2:**
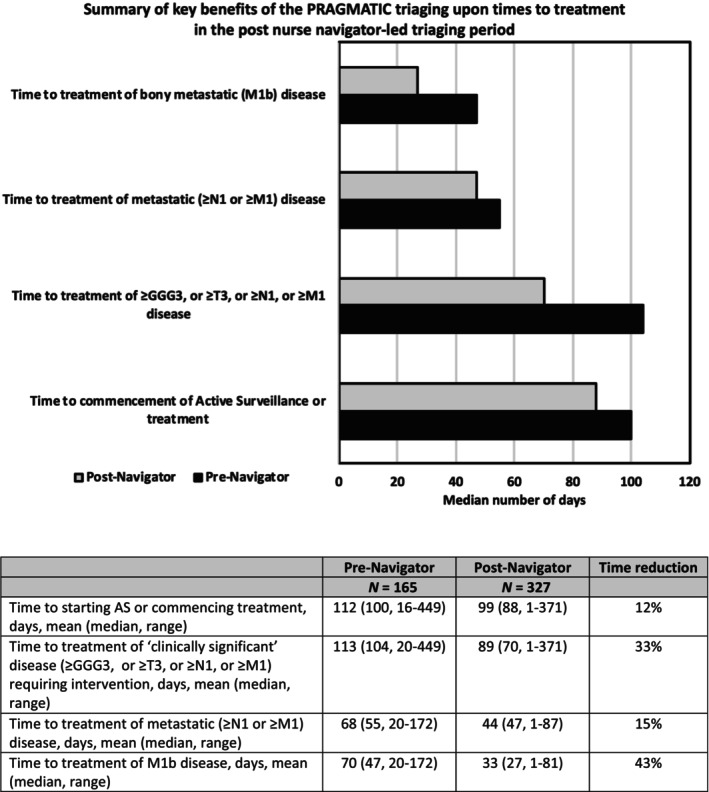
Summary of the key benefits of the PRAGMATIC triaging upon times to treatment for patients referred on the urgent suspected PCa pathway in the pre‐ and post‐nurse ‘navigator’ time periods. GGG, Gleason Grade Group.

Analysis of times to treatment, and features of these treatments, for patients referred on the urgent suspected PCa pathway in the pre‐ and post‐nurse navigator time periods is shown in Table 3. Similar proportions of biopsied patients were found to have any‐grade PCa in the pre‐navigator (68/84 [81%]) vs the post‐navigator (118/162 [72.8%]) cohorts (*P* = 0.16). Similar proportions of patients presented with PCa requiring treatment (defined as Gleason Grade Group ≥3, or T‐stage ≥3, or N‐stage ≥1, or M‐stage ≥1 disease; 49/75 [65.3%] pre‐ vs 72/128 [56.3%] post‐navigator, *P* = 0.203), or with metastatic PCa (defined as N‐stage ≥1, or M‐stage ≥1 disease) (15/75 [20%] pre‐ vs 32/128 [25%] post‐navigator, *P* = 0.415), or with bone metastases (M1b disease; 11/75 [15%] pre‐ vs 18/128 [14%] post‐navigator, *P* = 0.905). The median time to starting AS, or to commencement of treatment, for any‐grade PCa was reduced from 112 to 99 days, although this did not reach statistical significance (*P* = 0.189; Table 3), demonstrating that the PRAGMATIC approach did not negatively impact low‐risk disease. The median time from referral to commencement of treatment for those patients who definitely need therapy (defined as Gleason Grade Group ≥3, or T‐stage ≥3, or N‐stage ≥1, or M‐stage ≥1 disease) was significantly reduced through use of the nurse navigator‐led PRAGMATIC triaging criteria (104 days pre‐ vs 70 post‐navigator, *P* = 0.013). The median time from referral to commencement of ADT for patients with M1b disease was observed to be significantly reduced in the period following nurse navigator oversight of the FDS clinical pathway (47 days pre‐ vs 27 post‐navigator, *P* = 0.031). The key benefits of the PRAGMATIC triaging upon times to treatment are summarised in Fig. 2.

**Table 3:** Analysis of times to treatment, and features of these treatments, for patients referred on the urgent suspected PCa pathway in the pre‐ and post‐nurse ‘navigator’ time periods.

Variable	Pre‐navigator, *N* = 165	Post‐navigator, *N* = 327	*P*
PCa T‐stage (including patients with a clinical diagnosis of PCa without biopsy), *n/N* (%)			
Tx	0	2/128 (1.6)	
T1	1/75 (1.3)	1/128 (0.8)	
T2	40/75 (53.3)	70/128 (54.7)	
T3	26/75 (34.7)	47/128 (36.7)	
T4	8/75 (10.7)	8/128 (6.2)	
PCa N‐stage, *n/N* (%)			
N0/Nx	65/75 (86.7)	98/128 (76.6)	
N1/N2	10/75 (13.3)	30/128 (23.4)	
PCa M‐stage, *n/N* (%)			
M0/Mx	62/75 (82.7)	106/128 (82.8)	
M1a	2/75 (2.7)	4/128 (3.1)	
M1b	11/75 (14.6)	18/128 (14.1)	
PCa management, *n/N* (%)			
AS	9/76 (11.8)	29/125 (23.2)	
Watchful waiting	6/76 (7.9)	11/125 (8.8)	
Radical prostatectomy	18/76 (23.7)	15/125 (12)	
Radiotherapy (±concomitant ADT)	31/76 (40.8)	43/125 (34.4)	
Brachytherapy	0	1/125 (0.8)	
Focal therapy	1/76 (1.3)	1/125 (0.8)	
ADT	11/76 (14.5)	25/125 (20)	
Time to starting AS or commencing treatment, days, mean (median, range)	112 (100, 16–449)	99 (88, 1–371)	0.189*
PCa requiring treatment (≥GGG3, or ≥T3, or ≥N1, or ≥M1 disease), *n/N* (%)	49/75 (65.3)	72/128 (56.3)	0.203^†^
Time to treatment of ‘clinically significant’ disease (≥GGG3, or ≥T3, or ≥N1, or ≥M1) requiring intervention, days, mean (median, range)	113 (104, 20–449)	89 (70, 1–371)	**0.013** *
Metastatic PCa (≥N1 or ≥M1 disease), *n/N* (%)	15/75 (20)	32/128 (25)	0.415^†^
Time to treatment of metastatic (≥N1 or ≥M1) disease, days, mean (median, range)	68 (55, 20–172)	44 (47, 1–87)	0.157*
Bone metastases from PCa (M1b disease), *n/N* (%)	11/75 (15)	18/128 (14)	0.905^†^
Time to treatment of M1b disease, days, mean (median, range)	70 (47, 20–172)	33 (27, 1–81)	**0.031** *
Lost to follow‐up/non‐compliant, *n*	1	6	

Bold values statistically significant at *P* < 0.001. GGG, Gleason Grade Group.

* Independent‐samples Mann–Whitney *U* test.

^†^ Pearson chi‐square.

## Discussion

The current FDS targets for diagnosis and treatment of PCa do not emphasise the need to rapidly diagnose and treat the most clinically significant forms of this common and ubiquitous malignancy, nor do they provide guidance regarding risk‐stratification of the highest‐risk (locally advanced and metastatic) cases. This provides healthcare providers and patients with a dilemma, given the ever‐increasing number of men referred for specialist investigation based on the suspicion of a PCa diagnosis. The number of patients on the FDS pathway at our institution continues to increase year‐on‐year, and the twin challenge of demand vs capacity, and the need to diagnose and treat the most high‐risk cases as quickly as possible without negatively impacting the low‐risk cases, is increasingly problematic. Whilst there have been several important clinical developments in the investigation and treatment of PCa in the last decade that have improved patient care [8], such as pre‐biopsy MRI [7, 9], targeted biopsy via TRUS or LATP [10, 11], and molecular staging imaging with PSMA‐PET‐CT [12], these have added time to the length of the clinical pathway for many patients, making it difficult to achieve arbitrary FDS targets. There is also the concern that the ever‐increasing need for resources to be directed to the FDS pathway for PCa may have a negative impact on the resources available to investigate and treat other urological malignancies such as bladder and renal cancer, and/or other time‐sensitive benign urological conditions such as ureteric stone disease. Given this clinical dilemma, we developed an in‐house bespoke PRAGMATIC triaging system, with which to expedite the steps in the rapid investigation pathway for those patients who may apparently have high‐risk PCa. We appointed a nurse navigator to oversee and manage the journey of patients on the pathway, with the ability to expedite investigations in those patients with high‐risk features, whilst ensuring that other low‐risk patients are not disadvantaged. We herein describe the results of such an approach at our institution. Despite there being a year‐on‐year increase in the number of patients on the FDS pathway in 2024 (post‐navigator) vs 2022 (pre‐navigator), we observed significant reductions in the time from referral to multiple points on the suspected PCa investigation and treatment pathway following triage by the nurse navigator. Encouragingly, we observed significant reductions in time from referral to treatment for the high‐risk cases of this malignancy, including those with high‐risk localised, locally advanced, and metastatic disease. Moreover, this improvement was not to the detriment of other low‐risk PCa cases, where times to diagnosis were not delayed because of the prioritisation and expeditious investigation of the high‐risk cases. It is noteworthy that the PRAGMATIC nurse navigator‐led triage system provided greatest benefit for patients with bony metastatic disease, with an observed reduction in the time to treatment from a median of 47 to 27 days, without negatively impacting the time to commencement of AS or all treatment options for all PCa risk types (median 100 vs 88 days, *P* = 0.189). Based on the results of this study, we would encourage policymakers and guidelines committees to consider modifying the FDS targets to reflect the risk strata that are known to exist for this malignancy. We appreciate that all men referred for specialist investigations to identify, or hopefully exclude, a diagnosis of PCa will wish to have these investigations in as rapid a timescale as possible and will either want the reassurance that they do not have PCa (or perhaps that they have low‐risk disease suitable for AS), or in the case of high‐risk disease will wish to start treatment as quickly as possible. However, the clinical priority ought to be to diagnose and commence treatment most rapidly in those cases of this heterogenous malignancy where we believe time makes a difference to clinical outcomes and prognosis, i.e., high‐risk localised, locally advanced, and metastatic disease cases. We have observed that use of a nurse navigator to oversee and manage the journey of patients on the FDS pathway, combined with in‐house adoption of the PRAGMATIC criteria by all members of the clinical team, can be associated with improved timescales for investigation and commencement of treatment for those with high‐risk PCa subtypes, without unduly compromising the timescale for investigation of other patients on the pathway.

This study has several limitations. First, it investigated two randomly selected 3‐month time periods before and after the use of the navigator and the PRAGMATIC criteria. Second, there are no data to inform us if the reduction in time to diagnosis and treatment of the high‐risk cases improved clinical outcomes for these patients. Third, we do not have any patient‐reported outcomes or results regarding their experience of the clinical pathway, which would be helpful to know for both those patients with high‐risk disease, and for those with benign findings or with low‐risk PCa suitable for AS. Fourth, we do not know if the impetus from this study was maintained, and if so, what additional resource may be necessary in order to maintain the anticipated benefit for the patients with high‐risk disease on the FDS pathway. Our results do not include a formal health economics evaluation, which would be helpful if included in a future study. However, the cost of a full time Band 6 nurse navigator is approximately £40 000–£45 000 per annum. This may to an extent be offset by reduced penalties for pathway breaches, improved clinic utilisation, and avoidance of unnecessary scans or biopsy procedures (with resultant benefits to costs [17]), and the reduction in time‐to‐treatment of localised and metastatic PCa may lead to improved progression‐free and PCa‐specific survival, although further research is needed to explore this possibility. It is likely that additional resource may be needed beyond the reported study period, and beyond our PRAGMATIC triaging and the nurse navigator, such as consultant‐led clinics and delivery of treatments such as radiotherapy and surgery. A logical future direction could be to incorporate use of artificial intelligence (AI) into the PRAGMATIC triaging and streamlining, along with AI‐based reporting of MRI scans and prostate biopsies, as has recently been demonstrated in the PCa MDT setting [18].

In conclusion, we observed that nurse navigator‐led triaging and stratification of the most clinically urgent suspected PCa cases, and use of in‐house PRAGMATIC criteria to expedite investigation and treatment for high‐risk cases, was associated with improved times to imaging, biopsy diagnosis, and treatment for those patients on the pathway in most need, without apparent detriment to other patients.

## Disclosure of Interests

Richard J. Bryant, Alastair D. Lamb, and Tom Leslie have received support from BXT Accelyon to attend LATP biopsy training provided by Guys’ Hospital, London, UK. Richard J. Bryant has received research funding from Cancer Research UK, Prostate Cancer UK, and National Institute for Health Research (NIHR‐HTA), has received honoraria from Koc University (Istanbul, Turkey), has received support for travel to the FOCAL+ 2025 meeting, is a member of the STAMINA Clinical Trial Steering Committee, and is an unpaid trustee of UCARE (Oxford). Alastair D. Lamb has received research funding from Cancer Research UK and the John Fell Charitable Trust, consulting fees from Alpha Sights, GenesisCare, and Astellas, lecture honoraria from University of Michigan, Koc University (Istanbul), Sri Lankan Medical Association, 10X Genomics, and nanoString, payment for expert testimony from each of Wollens, Irwin Mitchell, Goodlaw and Glynns Solicitors, support for meetings from Astellas, BXT Accelyon, and Koelis, is on the Data Monitoring Safety Committee of the Neurosafe PROOF Trial, is a member of the Advisory Boards of Movember and 3P (Institute of Cancer Research), has received training support and trial of devices from BXT Accelyon, BK Medical Devices, Koelis, Leapmed, and Intuitive Surgical, and is section editor of the *BJUI*. Freddie C. Hamdy has received research grants from Cancer Research UK and Prostate Cancer UK, consulting fees from Intuitive Surgical, honoraria from Eureka Sri 2023 payment for expert testimony from Hamad Medical Corporation, and support for meetings or travel (2024) from the University of Gothenberg, EMUC, Chongqing Haifu Medical Technology, Royal College of Physicians and Surgeons of Glasgow, UROART (Basel), and FOCAL+ 2023, and is Editor‐in‐Chief of the *BJUI*. Clare Verrill is partly funded by the NIHR Oxford Biomedical Research Centre, is chair of the British Association of Urological Pathologists, is principal investigator of a study evaluating Paige Prostate AI and has received research funding from Prostate Cancer UK. All other authors declare no competing interests.

## Supporting information


**Fig. S1.** A visual detail of the nurse navigator‐led triaging of the clinical pathway for patients urgently referred with suspected PCa.
