# Supplementary material for: The PRAGMATIC pathway ‐ PRostate cancer diAGnosis and MAnagement Triage In Clinical care
Source: BJU Int. 2026 Feb 26;137(5):849–57. doi: 10.1111/bju.70191 (PMC13071532; doi:10.1111/bju.70191)
Supplement: Supplementary file 1 — Fig. S1. A visual detail of the nurse navigator‐led triaging of the clinical pathway for patients urgently referred with suspected PCa. [file BJU-137-849-s001.docx]

**
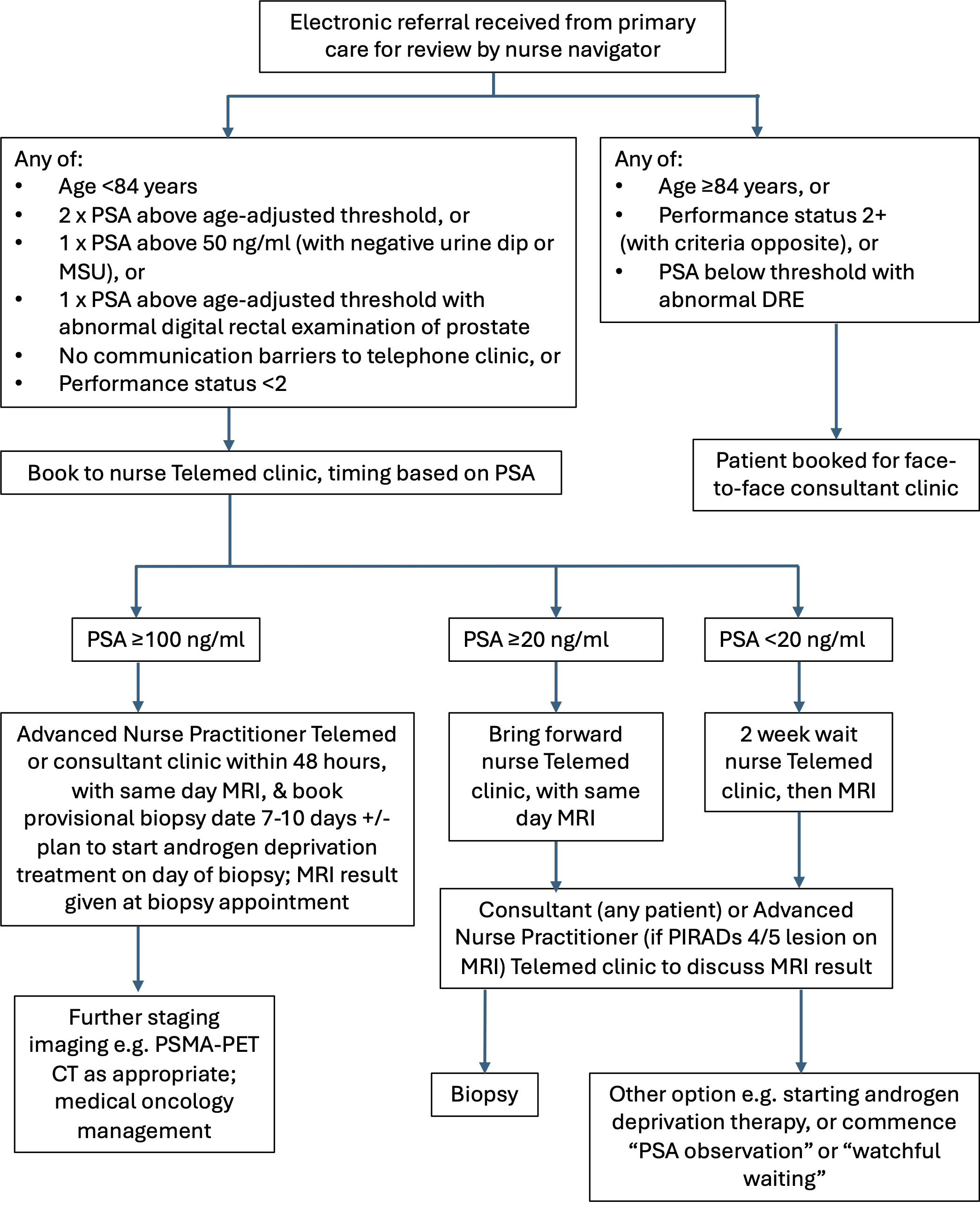
**

**Figure S1.** A visual detail of the nurse navigator-led triaging of the clinical pathway for patients urgently referred with suspected prostate cancer.
